# PDGF-D gene polymorphism is associated with increased cardiovascular mortality in elderly men

**DOI:** 10.1186/s12881-016-0325-z

**Published:** 2016-09-01

**Authors:** Urban Alehagen, Renate S. Olsen, Toste Länne, Andreas Matussek, Dick Wågsäter

**Affiliations:** 1 Department of Cardiology, University Hospital of Linköping, SE-581 85 Linköping, Sweden; 2 Department of Medical and Health Sciences, Linköping University, Linköping, Sweden; 3 Division of Drug Research, Department of Medical and Health Sciences, Faculty of Health Sciences, Linköping University, Linköping, Sweden; 4 Department of Laboratory Services, County Hospital Ryhov, Jönköping, Sweden; 5 Department of Medical and Health Sciences, Division of Cardiovascular Medicine/Physiology, University of Linköping, 581 85 Linköping, Sweden; 6 Department of Medical and Health Sciences, Division of Drug Research/Clinical Pharmacology, University of Linköping, 581 85 Linköping, Sweden

**Keywords:** PDGF-D, rs974819, Genotypes, Gender, Prognosis, Cardiovascular risk

## Abstract

**Background:**

Platelet-derived growth factor (PDGF) D has been reported to be active in fibroblasts, and in areas of myocardial infarction. In this longitudinal study we evaluated the association between PDGF-D polymorphism and cardiovascular mortality, and attempted to discover whether specific genotype differences regarding risk could be observed, and if gender differences could be seen.

**Methods:**

Four hundred seventy-six elderly community participants were included in this study. All participants underwent a clinical examination, echocardiography, and blood sampling including PDGF-D single nucleotide polymorphism (SNP) analyses of the rs974819 A/A, G/A and G/G SNP. The follow-up time was 6.7 years.

**Results:**

No specific genotype of rs974819 demonstrated increased cardiovascular mortality in the total population, however, the male group with genotypes A/A and G/A demonstrated an increased risk that persisted in a multivariate evaluation where adjustments were made for well-known cardiovascular risk factors (2.7 fold compared with the G/G genotype). No corresponding finding was observed in the female group.

**Conclusion:**

We report here for the first time that the genotypes G/A or A/A of the SNP rs974819 near PDGF-D exhibited a 2.7 fold increased cardiovascular mortality risk in males. Corresponding increased risk could not be observed in either the total population and thus not in the female group. However, the sample size is was small and the results should be regarded as hypothesis-generating, and thus more research in the field is recommended.

## Background

The platelet-derived growth factor (PDGF) family presently consists of four members (PDGF-A, PDGF-B, and the recently discovered PDGF-C and PDGF-D) [1–3]. The PDGF family perform a multitude of functions such as vasodilation, regulation of erythropoiesis, and wound repair [4], but are also major mitogens for fibroblasts and smooth muscle cells, and are involved in the atherosclerotic process in the body [5, 6]. PDGF-D has also been reported active in conjunction with various tumors, such as prostate cancer [7], and soft tissue sarcomas [8].

After a myocardial infarction, increased levels of PDGF-D in the infarcted tissue have been reported, a fact that has been interpreted to indicate involvement of PDGF-D in the repair process [9]. It has also been shown that the PDGF-D expression is increased late after a myocardial infarction, resulting in increased production of interstitial fibroblasts [9]. PDGF-D has also been shown to up-regulate the expression of matrix metalloproteinase-1 (MMP-1), MMP-2 and MMP-9 in a rat model [10]. In a genome-wide association study of risk loci for coronary heart disease in Europeans and in South Asians, Peden et al. showed that rs974819 of PDGF-D was one of the top 10 % of risk loci for coronary heart disease [11]. This is in congruence with another large-scale genome association study [12]. However, little has been reported on rs974819 genotypes and risk of cardiovascular death, and there has been no study of gender differences regarding cardiovascular mortality.

The aim of this study was to evaluate the distribution of the three genotypes G/G, G/A and A/A of rs974819 to see if any differences in cardiovascular mortality could be registered in the three groups, and finally if gender differences could be noted.

## Methods

### Patient population

The study population, were all part of a longitudinal epidemiological study focusing on cardiovascular risk factors, and were invited to participate in the present sub-study conducted from 13th January 2003 to 18th June 2005.

In 1999, all inhabitants aged 64–82 in a rural municipality in south east Sweden were invited to take part in a longitudinal study. In order to minimize bias in the selection process of obtaining a representative population of elderly persons, all those living in the municipality in a specific age span were invited to participate in the longitudinal project. Of 1162 subjects in the age 65–82, 876 (75 %) agreed to participate in the primary project. When the present follow-up study was conducted four to five years later 675 agreed to participate, and 476 agreed to deliver blood samples, and could present Doppler-echocardiographical examinations with acceptable quality. All participants underwent a clinical examination, ECG recording, and Doppler-echocardiography, and blood samples were collected. The New York Heart Association (NYHA) functional classification was evaluated by the including physician based on the patient information. All participants gave their written informed consent, and the study was conducted in accordance with the Declaration of Helsinki principles. The study protocol was approved by the Regional Ethical Review Board of Linköping, Sweden (Dnr 95044). The mortality information was obtained after permission for this was approved, from autopsy reports or from the National Board of Health and Welfare in Sweden, which registers all deaths. The causes of death are mainly based on death certificates with about 10 % based on autopsies, with potentially limited reliability. A recent study suggests a 55 % accuracy of death certificates in cardiovascular disease in a Swedish community [13].

The main project has been presented in several previous publications [14–25].

### Co-morbidity

Diabetes mellitus was defined as a previous diagnosis with on-going treatment, or a fasting blood glucose ≥ 7 mmol/L measured at one single occasion. Hypertension was defined as a blood pressure of more than 140/90 mm Hg measured in the right arm with the patient in the supine position after at least 30 min rest. Patients were defined as hypertensive if they had previously been diagnosed with hypertension and were receiving antihypertensive medication. Ischemic heart disease was defined as a history of angina pectoris/myocardial infarction or ECG-verified myocardial infarction. Heart failure was defined as a previous diagnosis with on-going treatment, or symptoms/signs of heart failure and objective demonstration of reduced cardiac function in terms of impaired cardiac function on echocardiography, or increased plasma concentration of pro B-type natriuretical peptide (NT-proBNP) 1–76.

### Echocardiography

Echocardiography examinations were performed using an Accuson XP-128c with the patients in a supine left position. Values for systolic function expressed as left ventricular ejection fraction (EF), were categorized into four classes with interclass limits of 30, 40 and 50 %. Normal systolic function was defined as EF ≥ 50 %.

### Biochemical analyses

All blood samples were obtained while the patients were at rest in a supine position, and all samples were collected in pre-chilled plastic Vacutainer tubes (Terumo EDTA K-3). Plasma was prepared by centrifugation at 3000 g for 10 min at 4 °C. All samples were stored at −70 °C until analysis. None of the samples were thawed more than twice.

NT-proBNP was measured in the Elecsys 2010 platform (Roche Diagnostics, Mannheim, Germany). The total CV was 4.8 % at 26 pmol/L and 2.1 % at 503 pmol/L (*n* = 70) at our laboratory.

### Genotype determination

Genomic DNA was isolated from peripheral blood using the QIAmp DNA Mini Kit (QIAGEN, Hilden, Germany), following the manufacturer’s instructions.

A LightSNiP genotyping assay (TIB Molbiol GmbH, Berlin, Germany) was used for analysis of PDGF-D (rs974819) genotypes. DNA was mixed with Reagent Mix, LightCycler® FastStart DNA Master HybProbe (Roche Diagnostics) and amplified using the LightCycler® 480 Real-Time PCR System (Roche Diagnostics).

### Statistical methods

Descriptive data are presented as percentages or mean and standard deviation (SD). Comparative analyses were performed using the Student unpaired two-sided *T*-test, whereas the chi-square test was used for discrete variables. Both univariate and multivariate Cox proportional hazard regression analyses were used to analyse and illustrate the risk of mortality during the follow-up period, where both all-cause mortality and cardiovascular mortality were analysed. Kaplan-Meier graphs were used to illustrate cardiovascular mortality as a function of follow-up time. Censored patients were patients who were still alive at the end of the study period or who had died of other causes than cardiovascular disease. Completed patients comprised those who had died due to cardiovascular disease. In the multivariate multivariable regression model adjustment were made for the following co-variates; age, diabetes, hypertension, ischemic heart disease, body mass index (BMI), Hb < 120 g/L, estimated glomerular filtration rate (eGFR) < 60 mL/min, ACE- inhibitors/Angiotensin receptor blockers, beta blockers, EF < 40 %, and s-cholesterol.

A *P*-value < 0.05 was considered statistically significant. All data were analysed using standard software packages (Statistica v. 12.0, Statsoft Inc, Tulsa, OK, USA).

## Results

The basal characteristics of the 476 participants divided into the two genders are presented in Table 1. The female group had a higher BMI (27.6 vs. 26.7; *P* = 0.02), and also a higher proportion of anaemia, here defined as Hb < 120 g/L (35 vs. 16; *P* = 0.003), and a higher mean HDL (1.6 mmol/L vs. 1.3 mmol/L; *P* < 0.0001). The male group exhibited a significantly higher ratio of impaired systolic cardiac function, here defined as EF < 40 % (27 vs. 9; *P* = 0.003) (Table 1). In the total study population, 75 % had diagnosed hypertension, 26 % were on treatment with ACE-inhibitors/Angiotensin receptor blockers, and 35 % were on treatment with beta-blockers. The mean follow-up time was 6.7 years. During the follow-up period there was an all-cause mortality of 22.3 % (106 out of 476), and of those 13.0 % (62 out of 476) suffered a cardiovascular mortality.

**Table 1:** Basal characteristics of the total study population and divided into genders

Variables	Total population	Females	Males	*p*-value
N	476	234	242	
Age, mean (SD)	77 (3.4)	77 (3.7)	77 (3.2)	0.81
BMI, mean (SD)	27.2 (4.3)	27.6 (5.1)	26.7 (3.3)	*T* =2.40; *P* = *0*.02
IHD, n (%)	105 (22.1)	42 (17.9)	63 (26.0)	X^2^ = 4.52;*P* = 0.03
Hypertension, n (%)	359 (75.4)	185 (79.1)	174 (71.9)	0.07
Diabetes, n (%)	154 (32.4)	68 (29.2)	86 (35.5)	0.13
ACEI/ARB, n (%)	125 (26.3)	61 (26.1)	64 (26.4)	0.73
Beta blockers, n (%)	168 (35.3)	78 (33.3)	90 (37.2)	0.38
Hb < 120 g/L, n (%)	51 (10.7)	35 (15.0)	16 (6.6)	X^2^ = 8.66; *P* = 0.003
HDL, mmol/L mean, (SD)	1.5 (0.4)	1.6 (0.4)	1.3 (0.3)	*T* =7.82;
				*P* < 0.0001
CRP, mg/L, mean (SD)	11.0 (5.1)	11.0 (5.3)	10.8 (5.0)	0.68
EF < 40 %, n (%)	36 (7.6)	9 (3.8)	27 (11.2)	X^2^ = 9.10;
				*P* = 0.003
NT-proBNP ng/L, mean, (SD)	188 (319)	174 (200)	200 (395)	0.37

Notes: *ACEI/ARB* angiotensin converting enzyme inhibitor/Angiotensin receptor blocker, *BMI* body mass index, *CRP* C-reactive protein, *EF* ejection fraction, *eGRF* estimated glomerular filtration rate, *HDL* high density lipoproteins, *IHD* ischemic heart disease, *NT-proBNP* N-terminal fragment of proBNP

Note: P-value is based on comparison between females and males

### Mortality and genotypes PDGF-D rs974819

The genotype distribution and the distribution of all-cause and cardiovascular mortality within the different genotypes in the total study population and in the two genders are presented in Tables 2 and 3. The frequency of the A/A genotype was 8.8 % in the total population, similar to a European population in the HapMap CEU reporting of 7.3 %.

**Table 2:**
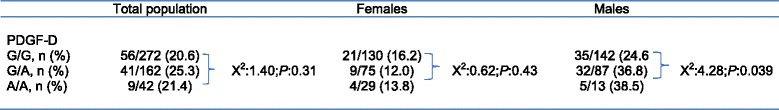
All-cause mortality in the study population distributed into the three analyzed genotypes of the PDGF-D gene

Note; n indicates the amount of all-cause mortality in relation to the total amount in the actual group

Note: in the X^2^-test, comparison between G/G versus G/A or A/A has been performed, as indicated in the brackets

**Table 3:**
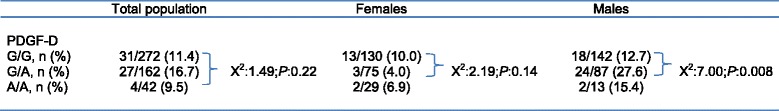
Cardiovascular mortality in the study population distributed into the three analyzed genotypes of the PDGF-D gene

Note; n indicates the amount of cardiovascular mortality in relation to the total amount in the actual group

Note: in the X^2^-test, comparison between G/G versus G/A or A/A has been performed, as indicated in the brackets

Intriguingly, the A/A frequency was more than twice as high among females compared to males (29/234; 12.4 % vs. 13/242; 5.4 %) (Tables 2 and 3).

**Fig. 1:**
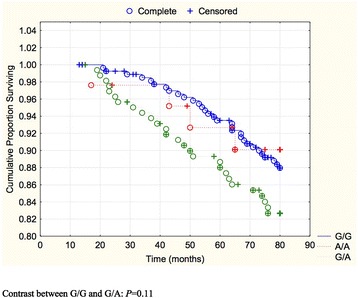
Kaplan-Meier graph illustrating cardiovascular mortality distributed into the genotypes G/G, G/A, and A/A of the SNP rs974819 in the total study population during 80 months of follow-up. Note: Censored participants were those still living at the end of the study period, or who had died of reasons other than cardiovascular disease. Completed participants were those who had died due to cardiovascular disease

The cardiovascular mortality of the three genotypes in the total study population during a follow-up period of 80 months was illustrated in a Kaplan-Meier graph (Fig. 1).

A power calculation indicated a sample size of males to 290 participants (alpha:0.05, beta: 0.2, power: 0.8), where 242 participants were recruited. For the female group, the required sample size was 790 participants, and where we reached 234 participants, thus a too small to reach a possible significance.

**Fig. 2:**
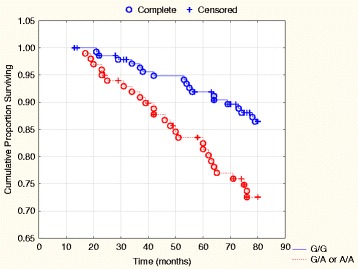
Kaplan-Meier graph illustrating cardiovascular mortality distributed into the G/A or A/A genotypes combined compared to the G/G genotype of the SNP rs974819 in the male population during 80 months of follow-up. Note: Censored participants were those still living at the end of the study period, or who had died of reasons other than cardiovascular disease. Completed participants were those who had died due to cardiovascular disease

The main finding is that the G/A and the A/A homozygotes amalgamated into one group among the males exhibit a higher incidence of both all-cause (*P* = 0.039) and cardiovascular mortality (*P* = 0.008) compared to the G/G genotype (Tables 2 and 3). This has been illustrated in a Kaplan-Meier analysis showing that in the male group, significantly more patients suffered cardiovascular mortality among the G/A heterozygotes or the A/A homozygotes, compared to the G/G homozygotes (Fig. 2).

To evaluate increased cardiovascular mortality risk of the G/A or A/A genotypes, a univariate Cox proportional hazard regression analysis was performed.

In the total study population, the G/A or A/A genotypes did not fulfil significant prognostic information (HR: 1.39; *P* = 0.20 unadjusted; HR: 1.35; *P* = 0.24, when adjusted for gender and age). However, in the male group a significant increase in cardiovascular mortality risk of the G/A or A/A variant could be demonstrated in the univariate Cox proportional hazard regression (HR: 2.25; CI 95 % 1.23–4.10; *P* = 0.008). In a multivariate model including clinical variables known to influence cardiovascular risk, the G/A or A/A genotypes still exhibited significant and independent prognostic information, with a 2.7 fold increased cardiovascular mortality risk for the male participants with those genotypes (Table 4).

**Table 4:** Multivariate Cox proportional hazard regression illustrating risk of cardiovascular mortality in the population divided into the two genders, and with a follow-up period of 80 months

Variables	Females			Males		
	Hazard ratio	95 % confidence interval	*P*-value	Hazard ratio	95 % confidence interval	*P*-value
Age	1.16	0.99–1.37	0.07	1.16	1.04–1.28	0.006
Diabetes	2.66	0.92–7.65	0.07	0.98	0.51–1.89	0.95
Hypertension	1.47	0.40–5.36	0.56	1.39	0.69–2.83	0.36
IHD	2.18	0.64–7.42	0.21	0.79	0.37–1.70	0.54
BMI	1.06	0.96–1.17	0.22	0.89	0.80–0.99	0.04
Hb < 120 g/L	2.12	0.71–6.35	0.18	1.35	0.43–4.23	0.61
eGFR < 60 mL/min	3.06	0.78–12.00	0.11	0.82	0.41–1.64	0.58
ACEI/ARB	0.96	0.34–2.71	0.93	1.04	0.50–2.15	0.92
Beta blockers	0.91	0.32–2.55	0.86	1.07	0.53–2.19	0.84
EF < 40 %	1.08	0.20–5.85	0.93	2.38	0.98–5.77	0.05
s-Cholesterol	0.91	0.58–1.41	0.66	0.96	0.70–1.33	0.83
PDGF-D G/A or A/A	0.36	0.12–1.05	0.06	2.72	1.39–5.32	0.003

Notes: *ACEI/ARB* ACE-inhibitors, or Angiotensin receptor blockers, *BMI* body mass index, *EF* ejection fraction based on echocardiography, *IHD* ischemic heart disease

Note: eGFR was based on the MDRD formula

**Fig. 3:**
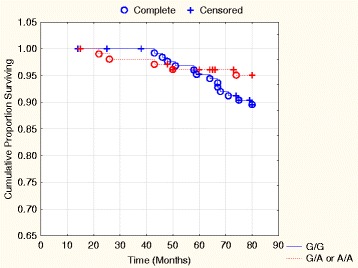
Kaplan-Meier graph illustrating cardiovascular mortality distributed into the G/A or A/A genotypes combined compared to the G/G genotype of the SNP rs974819 in the female population during 80 months of follow-up. Note: Censored participants were those still living at the end of the study period, or who had died of reasons other than cardiovascular disease. Completed participants were those who had died due to cardiovascular disease

In the female group the G/A or A/A genotypes did not exhibit significant prognostic information regarding cardiovascular mortality in the univariate Cox proportional hazard regression analysis (HR: 0.48; *P* = 0.16). A Kaplan-Meier graph illustrates the cardiovascular mortality as a function of follow-up time of the G/A or A/A genotypes in comparison to the G/G genotype of the female group could be seen in Fig. 3, where no difference between the three genotypes could be demonstrated.

## Discussion

Recently a discussion on how to optimally handle patients with increased risk of cardiac events has been launched, partly due to restricted health resources. The inclusion of genetic markers into clinical routines has also been discussed. In this study, PDGF-D has been evaluated, partly because data in the literature indicate that there is an association between the SNP rs974819 near PGDF-D, and coronary artery disease [12]. Another reason is that PDGF-D is reported to be active in tissue with active fibroblasts and smooth muscle cells, especially in cardiac tissue [6].

In the present study the G/G, G/A and A/A genotypes of rs974819 have been evaluated in an elderly population. The population evaluated consisted of subjects from a rural Swedish municipality, and thus could be regarded as representative of an elderly community population. The differences between males and females regarding basal characteristics were as expected (Table 1), with more impaired cardiac function in the male group and more patients with increased BMI in the female group. The A/A genotype of rs974819 is by far the rarest and was therefore difficult to evaluate from a risk perspective with the small number of subjects included. Therefore, the A/A and the G/A genotypes have been amalgamated into one group for comparison with the G/G genotype in the multivariate risk evaluation (Table 4), as well as in Figs. 2 and 3. Thus, in the evaluations it seems that the male population with the G/A or A/A genotypes exhibits higher mortality risk, especially cardiovascular risk. However, a corresponding increased risk in the total population, or in the female group could, however, not be demonstrated. That males have a higher cardiovascular risk, especially in ischemic events is well-known [26, 27], even though the female group catch up at higher age [28]. It is therefore interesting to note the persisting difference in cardiovascular risk between the three genotypes of rs974819 in this elderly population. Even when adjustments have been made for well-known clinical variables influencing cardiovascular risk, the G/A or A/A genotypes were associated with a 2.7 fold increased cardiovascular risk in the male population.

Zhou et al. presented an evaluation of 161 Chinese patients with coronary heart disease compared to 112 controls, where different genotypes and alleles of rs974819 were compared. They presented that rs 974819 was associated with coronary heart disease and that the minor allele G was associated with a 45 % lower risk of coronary heart disease compared to the A allele [29]. In their study a greater proportion of the A allele could be seen in the Chinese study population (0.68), as also reported from South Asian studies (0.35), compared to the European population (0.29), and also demonstrated by Peden et al. [11]. In our population we could also demonstrate a smaller proportion of the A/A genotype of rs974819 compared to the other two genotypes. However, comparisons were not straightforward due to the large difference in allele frequency. Zhou presented data indicating a higher incidence of coronary heart disease in females with the A allele, but no difference could be demonstrated in males regarding coronary heart disease between the G and the A allele. In the present study the male group with the G/A or A/A genotypes demonstrated the highest cardiovascular mortality risk, again probably as a result of ethnic differences, but possibly also as a result of different lifestyles between the males and female populations that might facilitate the disease progress. Comparing with the study by Zhou et al., the present study applied a more definite hard end-point; cardiovascular mortality compared to coronary heart disease, as used by Zhou et.al. The difference in coronary heart disease/mortality between females and males as reported in the present study compared to the data presented by Zhao et al. from a Chinese population could be partly explained by that female patients seems to be more severely diseased and have a worse prognosis compared to males [30]. In a recent publication by Zimerman et al. where four different trials on acute coronary syndrome was evaluated, female gender was one independent variables for severe adverse events [31]. In a substudy of the Valsartan in Acute Myocardial Infarction Trial (VALIANT), the same messages could be demonstrated, even if the size of male group included with myocardial infarction was more than double compared to the female size [32].

The present study has, however, applied an unusually long follow-up time to evaluate the end-result of the coronary heart disease process, and by applying a perspective over more than 6 years it might be possible to identify possible differences in risk also in this relatively small population. This might result in that more males are identified compared to females.

The studied population was elderly, and those at high risk had probably already died. However, in spite of this it was possible to demonstrate differences in cardiovascular mortality between genotypes.

Again, however, the obtained results of the present study should be interpreted with caution, and should be regarded as hypothesis-generating.

## Limitations

Several limitations could be identified. The first limitation is the limited size of the study sample, which influenced the statistical evaluation.

The high age of the included population could also be regarded as a limitation, as it is not clear if the result could be extrapolated into younger subjects. The genotypes do not change with age, but there might be other aging processes and comorbidities that, together with a given genotype, might activate disease processes that results in increased risk.

Finally, the included population consisted of a homogenous population of Caucasians, so it is difficult to draw conclusions for other population groups as the literature demonstrates ethnic differences in this respect [11].

In conclusion, the presented study should therefore be regarded hypothesis- generating, and therefore more research is needed.

## Conclusion

The present study evaluated the three genotypes G/G, G/A and A/A of the SNP rs974819 of PDGF-D in 476 elderly community living individuals. From this a more than 2.7 fold increased cardiovascular mortality risk of the G/A or A/A genotypes in the male group could be noted, but not in the female group. This information could, in a situation where health resources are restricted, be important in order to identify patients at high cardiovascular risk, and thereby motivate a tighter and more costly follow-up net for those high risk patients.

However, as the sample size was small, the results should be interpreted with caution, and this work should be regarded more as a hypothesis-generating study.

## Abbreviations

BMI: Body mass index
EF: Ejection fraction
eGFR: Estimated glomerular filtration rate
MMP: Matrix metalloproteinase
NT-proBNP: N-terminal fragment of pro B-type natriuretical peptide
NYHA: New York Heart Association functional classification
PDGF: Platelet-derived growth factor
SD: Standard deviation
SNP: Single nucleotide polymorphism
